# Toxin Induced Parkinsonism and Hospitalization Related Adverse Outcome Mitigation for Parkinson’s Disease: A Comprehensive Review

**DOI:** 10.3390/jcm12031074

**Published:** 2023-01-30

**Authors:** Kenneth R. Dalton, Charles J. Kidd, Nawaz Hack

**Affiliations:** 1 Department of Neurology, Walter Reed National Military Medical Center, Uniformed Services University of the Health Sciences, Bethesda, MD 20814, USA; 2 Department of Neurology, UTRGV Institute of Neuroscience, UTRGV School of Medicine, Harlingen, TX 78550, USA

**Keywords:** Parkinson’s disease, toxins, Parkinsonism, neurologic emergencies, pearls

## Abstract

Patients with Parkinson’s disease admitted to the hospital have unique presentations. This unique subset of patients requires a multidisciplinary approach with a knowledge-based care team that can demonstrate awareness of complications specific to Parkinson’s disease to reduce critical care admissions, morbidity, and mortality. Early recognition of toxic exposures, medication withdrawals, or medication-induced symptoms can reduce morbidity and mortality. This review can assist in the critical assessment of new or exacerbating Parkinson’s disease symptoms.

## 1. Introduction

Parkinson’s disease (PD) remains the second- fastest growing movement disorder, with 7–10 million cases worldwide generating a financial burden of nearly $52 billion per year in the United States [1]. In a 2012 review, 45% of PD patients visit the emergency room (ER) annually, with 28% of these patients subsequently being admitted, making PD patients 1.4–1.5 times more likely to be hospitalized than age- and sex-matched controls [2,3]. Greater than 50% of the patients admitted are considered advanced-stage PD [4,5]. Based on a 2020 meta-analysis of 7162 admissions, the most common etiologies are infection (22%), worsening PD motor features (19%), falls/fractures (18%), cardiovascular comorbidities (13%), neuropsychiatric complications (8%), and gastrointestinal dysfunction (7%) [3]. A 2016 epidemiologic review of PD inpatient stays demonstrated the median age of patients admitted from 2002 to 2011 was 89.9 years, with a disposition to home (32.6%), facility (62.9%), or death (3.9%) [6]. Patients with PD also experience more perioperative complications, with PD being an independent risk factor for increased length of stay and morbidity when undergoing elective surgery, such as Deep Brain Stimulation (DBS) [2].

In a review of ER and hospital visits by PD patients (*n* = 1120), 152 (14%) had no regular caretaker with 2.3 ± 1.4 comorbidities, increasing their risk of medication mismanagement [5,7]. ER and hospital-related complications such as failure to recognize idiopathic PD, toxin-induced PD, and acute dopamine withdrawal, while considering increased risk for aspiration pneumonia, falls, timely medication administration, or avoidance of contraindicated pharmacotherapy are complex situations that can quickly result in care acuity escalation. In a review of 146 PD patients admitted to the hospital, 36% required Intensive Care Unit (ICU) admission for fever (34%), delirium (16%), falls (12%), encephalopathy (8%), gastrointestinal (6%), and dyskinesias (4%) [8]. We will review early recognition and recommendations for management to mitigate complications and improve outcomes. 

## 2. Toxins Induced Parkinsonism Presenting to the Emergency Room

### 2.1. Carbon Monoxide

Carbon monoxide (CO) is a gas that is rarely perceived by human senses. Inhalation, however, results in 40,000 annual ER visits and between 5000 and 6000 deaths [9]. It is estimated that 40% of patients who suffer from carbon monoxide poisoning will subsequently develop neurologic dysfunction [9]. A population-based cohort study (n = 9012) of patients exposed to carbon monoxide without comorbidities was at a 15.8-fold risk of developing PD [9]. A separate study of 242 patients demonstrated that 23 (9.5%) patients with carbon monoxide poisoning developed Parkinsonism within 2–26 (median = 4) weeks of exposure. 

#### 2.1.1. Pathophysiology

CO binds to hemoglobin (Hb) with a 250-fold greater affinity than oxygen [10]. CO binding results in decreased oxygen-carrying capacity of red blood cells, subsequently reducing oxygen delivery to the tissues [10]. CO subsequently inhibits mitochondrial respiration, halting oxidative phosphorylation, and decreasing Adenosine Triphosphate (ATP) production, which is most prominent in cerebral and cardiac tissue [10]. In the absence of ATP production, the electron transport chain (ETC) generates superoxide, resulting in cellular and tissue damage [10]. CO will also displace nitrous oxide (NO) from platelets, which binds with superoxide to produce peroxynitrite, resulting in additional mitochondrial inhibition and increased platelet activation [10]. Myeloperoxidase (MPO) and reactive oxygen species (ROS) are formed, which impact myelin basic protein, triggering a lymphocytic response and microglial activation [10]. Reduced cerebral oxygenation and increased mitochondrial inhibition result in anoxic brain injury, which is often delayed but can be rapid, resulting in death hours after CO poisoning [10]. Parkinsonism in the setting of CO poisoning is attributed to bilateral globus pallidus involvement [11]. While the exact pathogenesis of Parkinson’s induction from CO intoxication remains unclear, recent studies suggest it is a result of oxidative stress from structural damage, whether from reperfusion injury or prolonged ischemia [12].

#### 2.1.2. Clinical Presentation and Diagnosis

Acute presentation of CO poisoning manifests itself as headache, fatigue, nausea, emesis, cognitive impairment, chest discomfort, shortness of breath, lightheadedness, and potentially loss of consciousness [10]. Chronic exposure can result in fatigue, vertigo, paresthesia, abdominal pain, diarrhea, and polycythemia [10]. While acute presentations, with emergency medical services identifying exposure upon arrival, may be overtly diagnosed, chronic exposures may pose a diagnostic challenge. Given that conventional pulse oximetry may not identify COHb, clinical suspicion is necessary to accurately identify the presence of CO [10]. Non-specific neurologic manifestations of CO toxicity include changes in mood (anxiety, depression), cognitive dysfunction (memory), disequilibrium (vertigo), and motor deficits [10]. MRI of the brain may demonstrate bilateral globus pallidus T2 hyperintensities, in addition to diffuse atrophy with increased ventricular size and sulcal widening [13]. In severe exposures, the corpus collosum, internal capsule, external capsule, and subcortical white matter may also be affected [13].

Patients with CO exposure may present after 2–26 (median = 4) weeks with Parkinsonism and encephalopathy [11]. Parkinsonism manifestations include bradykinesia, masked faces, rigidity, and retropulsion with associated frontal release signs such as the grasp reflex or glabella sign [11]. While an intention tremor and disequilibrium are typically seen, a resting tremor is typically absent [11].

#### 2.1.3. Treatment

The standard of care is 100% normobaric oxygenation, though hyperbaric therapy has been utilized, and limited data exist to support hyperbaric over normobaric oxygen therapy [11]. Pharmacologic therapy is currently under investigation and may have pre-hospital applications, though none are currently Food and Drug Administration (FDA) approved therapies [11]. In our clinical experience, complete recovery can be achieved, though the prognosis is often dependent on exposure time and time to treatment. 

### 2.2. Manganese

Occupational history is a salient aspect of manganese toxicity evaluation, as exposure is common for intravenous (IV) drug abusers, welders, miners, steel workers, battery manufacturers, and fungicide production (Maneb) [14]. Grain, dried fruit, vegetables, nuts, and tea are the primary nutritional sources of manganese and may also be seen in patients on long-term parenteral nutrition [13]. Oral ingestion of food sources rarely causes toxicity, except in the setting of liver failure, which results in reduced excretion [13].

#### 2.2.1. Pathophysiology

Trivalent manganese is the reactive form that results in PD [13]. Trivalent manganese has a high affinity for neuromelanin, which can be found in high concentrations in the pars reticulata of the substantia nigra and basal ganglia, key anatomical aspects of PD [13]. Mitochondrial uptake inhibits oxidative phosphorylation and results in calcium accumulation [13]. Manganese also exhibits functional impairment of glutamate transport, increasing glutamate accumulation with subsequent apoptosis [13]. Autoregulation of dopamine release, in addition to the depletion of cerebral dopamine, results in amplified dopamine synthesis and release. Chronic sequelae result in neurotoxicity of the globus pallidus [13].

#### 2.2.2. Clinic Presentation and Diagnosis

At presentation, acute psychosis is often noted, with associated headache, vomiting, and hepatic dysfunction [13]. Psychosis will begin to resolve with the emergence of Parkinsonism, which will include steppage gait with dystonic features, imbalance, ataxia, and kinetic tremor [13]. The gait will be an important feature, as idiopathic PD presents with a shuffling gait [13]. Recognition of phenomenology and target occupational and exposure questioning are keys to early identification of manganese toxicity. Laboratory analysis and radiographic investigation may demonstrate elevated manganese in urine, serum, and whole blood [13,15]. MRI of the brain may demonstrate T1 hyperintensity in the caudate, putamen, and globus pallidus [13,15].

#### 2.2.3. Treatment

Removal of exposure is critical in the treatment of acute manganese toxicity [15]. Chelation therapy utilizing intravenous ethylenediaminetetraacetic acid (EDTA) increases manganese excretion, but has not demonstrated significant clinical improvement [14]. Patients may respond to dopaminergic therapy, though reports suggest it is less prominent when compared to idiopathic PD [14]. In our experience, prolonged manganese exposure results in persistent Parkinsonism that is not as responsive to medical therapy.

### 2.3. MPTP

1-methyl-4-phenyl-1,2,5,6-tetrahydropyridine (MPTP), formed as a byproduct of 1-methyl-4-phenyl-4-propionoxypiperidine (MPPP) is a meperidine analogue known to induce Parkinsonism in humans [16]. MPTP, sold under the guise of “Synthetic Heroin,” was originally identified in IV drug abusers [16]. No other sources of MPTP have been identified.

#### 2.3.1. Pathophysiology

MPTP induces a neurotoxic effect in the substantia nigra pars compacta because of Monoamine oxidase B (MOA-B) activity, generating 1-methyl-4-phenyl pyridine, inciting free radicals and oxidative stress [14]. 1-methyl-4-phenyl pyridine targets intracellular dopaminergic neurons by inhibiting Complex I of the ETC, resulting in endoplasmic reticulum stress and apoptosis [14]. MPTP is also a rare cause of Parkinsonism.

#### 2.3.2. Clinical Presentation and Diagnosis

Patients will present with rapid onset Parkinsonism manifesting as bradykinesia, and rigidity with a resting tremor in the setting of recent IV drug use. Clinical diagnosis is key, as there are no known biomarkers [14].

#### 2.3.3. Treatment

Acute onset MPTP Parkinson’s disease is a neurologic emergency requiring astute identification and rapid removal of the offending agent to minimize neuronal damage. Non-selective Monoamine Oxidase Inhibitor (selegeline) has been used to reduce neurotoxicity ameliorating clinical symptoms [16,17]. Symptomatic relief may be achieved with dopaminergic therapy [14]. This change is typically permanent in terms of developing PD-like symptoms, as documented clinically in past exposures [13].

### 2.4. Rotenone

Rotenone was discovered following exploration for a functional analogue of MPTP. Rotenone is primarily utilized as an agricultural insecticide and as an ingredient in home and pet products [18]. The naturally occurring plant species (timbo, barbasco, cub, haiari, and nekoe) have been employed as pesticides by indigenous groups [18]. As with MPTP, rotenone is a rare cause of PD; however, both serve as a reminder to consider exposures when hospitalizing patients with PD.

#### 2.4.1. Pathophysiology

Rotenone inhibits Complex I of the ETC, causing mitochondrial toxicity in the same pathway as MPTP, though microtubule destabilization has also been proposed as a mechanism [18]. Selective injury to the nigral dopaminergic neurons, in addition to cytoplasmic α-synuclein accumulation, induces motor and non-motor features of Parkinsonism [18]. Rotenone has a short half-life, without bioaccumulation, though animal models have demonstrated that brief exposures can result in progressive Parkinsonism.

#### 2.4.2. Clinical Presentation and Diagnosis

The diagnosis is based on clinical evaluation and exposure history. Human data is sparse, but based on rat models, repeated exposures are necessary to induce structural changes that result in the clinical manifestations of Parkinson’s disease [18,19]. Patients exposed to rotenone toxicity often present with conjunctivitis, dermatitis, pharyngitis, congestion, and vomiting when ingested [14]. Tachypnea may be present if the substance is inhaled [14]. Continued exposure will result in Parkinsonism with bradykinesia, postural instability, and rigidity [14]. No specific biomarkers are commercially available [14].

#### 2.4.3. Treatment

The offending agent should be promptly removed. Symptomatic management may be achieved with dopaminergic agents and an adenosine receptor agonist [14]. While not FDA approved, dietary phytocannabinoid has been shown to decrease neurotoxicity in rat models [14]. Clinically, Rotenone results in permanent PD-like symptoms that are minimally responsive to drug therapy.

## 3. Acute Cessation of High Dose Anti-Parkinson’s Therapy

### 3.1. Parkinsonism-Hyperpyrexia Syndrome

Parkinsonism-Hyperpyrexia Syndrome (PHS) is a result of acute dopamine agonist withdrawal in idiopathic PD [20]. PHS was first reported in 1981 due to neuroleptic exposure, with a notable withdrawal of dopaminergic therapy [21]. PHS is often misdiagnosed as Neuroleptic Malignant Syndrome (NMS), and may, at times, be an indistinguishable manifestation [18]. PHS is also referred to as acute akinesia, akinetic crisis, NMS-like syndrome, levodopa-withdrawal hyperthermia, dopaminergic malignant syndrome, and acute dopamine depletion syndrome [22,23].

PHS has been reported during withdrawal of non-dopaminergic medications used to treat idiopathic PD, such as anticholinergic therapy, amantadine, and tolcapone [20]. Failure to start dopaminergic therapy during hospitalization can result in PHS [23]. Cognizance of this syndrome is imperative following device-assisted therapy (i.e., bilateral subthalamic stimulation) to prevent rapid cessation or aggressive reduction of dopaminergic therapy [23].

#### 3.1.1. Pathophysiology

Acute dopaminergic therapy withdrawal impairs the anterior and posterior hypothalamic regulation of body temperature via normal thermoregulatory means [24]. Lack of dopamine results in blunted body cooling via peripheral vasodilation and the sudomotor (sweating) response [24]. Alteration of central serotonin metabolism and interference of the peripheral nervous system and central nervous system sympathetic outflow are suspected to be key contributors to PHS development [23]. Similar reactions occur with dopamine blockade (i.e., metoclopramide), which results in NMS [25].

#### 3.1.2. Clinical Presentation and Diagnosis

Symptom onset occurs between 18 h and 7 days after cessation of dopaminergic therapy and manifests as rigidity, tremor, and severe bradykinesia [23]. The tremor may be absent. Bradykinesia will eventually progress to akinesia and immobilization [23]. If unaddressed, pyrexia and a reduced level of consciousness will occur within 3–4 days of presentation, ultimately resulting in coma [23]. Patients eventually demonstrate autonomic dysfunction to include tachycardia, diaphoresis, and blood pressure instability [23]. Serum evaluation may demonstrate leukocytosis, abnormal liver function tests, and creatinine kinase (CK) elevation [23]. Severe sequelae include seizures, acute renal failure, rhabdomyolysis, disseminated intravascular coagulation, aspiration pneumonia, deep venous thrombosis, and pulmonary embolism [23]. The most commonly cited reason for dopamine withdrawal was reported to be a confused state or hallucinations [23].

The diagnosis is primarily clinical, though studies have shown reduced CSF concentrations of homovanillic acid (HVA), a metabolite of dopamine [23]. Decreased HVA is an expected biochemical response to the removal of dopamine, though lower levels were more noted in PHS [23].

#### 3.1.3. Treatment

Treatment hinges on early identification of recent dopamine cessation and reinitiating dopamine therapy [23]. In the event the patient has severe dysphagia or enteral tube administration is contraindicated, levodopa (L-dopa) can be administered IV at 50–100 mg over 3 h, repeating this 4 times daily until the patient can tolerate administration by mouth [23]. L-dopa dosing should be aligned with pre-PHS onset [23]. Bromocriptine (7.5–15.0 mg) may be utilized as an alternative therapy [23].

ICU monitoring for respiratory support or central venous pressure monitoring may be required [23]. Temperature management, as indicated, should include cooling blankets, IV fluid, and antipyretics [23]. Caution and monitoring for aspiration pneumonia should be taken into consideration [23]. Laboratory evaluation for renal dysfunction, coagulopathy, and rhabdomyolysis should be scheduled [23]. The patient should be monitored closely for the development of malignant hyperthermia, at which time Dantrolene sodium (10 mg/kg per day, divided into 3–4 doses) and bromocriptine (5–10 mg three times a day) should be administered [23].

Additional dopamine agonists, including ropinirole, pramipexole, transdermal rotigotine, and subcutaneous apomorphine, have been utilized [23]. There are no controlled studies demonstrating the efficacy of any specific medications in the treatment of PHS [23]. In our experience, a prompt restart of L-dopa therapy and transfer to the ICU results in reduced mortality and morbidity.

### 3.2. Dyskinesia-Hyperpyrexia Syndrome

Elevated levels of systemic L-dopa are known to induce dyskinesias. A term “dyskinetic storm”, or dyskinesia-hyperpyrexia syndrome, can result in a life-threatening presentation in patients undergoing L-dopa therapy [26]. This is an iatrogenic manifestation that can result in rhabdomyolysis or cardiac injury in patients with severe cardiomyopathy [26].

#### 3.2.1. Pathophysiology

L-dopa-induced dyskinesias are attributed to excess dopamine in the setting of dopaminergic nigro-striatal depletion [27]. As a result of dopamine depletion, discontinuous or pulsatile stimulation of dopaminergic receptors occurs [27]. Striatopallidal and thalamocortical alteration result in reduced mean discharge rate, subsequently altering typical firing patterns causing pathologic oscillatory activity which manifests as dyskinesias [27].

#### 3.2.2. Clinical Presentation and Diagnosis

Patients will present with persistent dyskinesia [27]. The diagnosis is dependent on the evaluation of L-dopa therapy and the correlation with symptom onset [27]. The diagnosis is primarily clinical. Creatinine kinase and myoglobin levels should be evaluated to determine if the patient is suffering from rhabdomyolysis [26,27]. If present, appropriate renal function screening should be undertaken [26,27]. Additionally, cardiovascular stress may result in cardiac strain, and appropriate evaluation with troponins or an ECG should be taken if clinically indicated [26].

#### 3.2.3. Treatment

Treatment of dyskinesia-hyperpyrexia syndrome should include antipyretic therapy, cooling as indicated, and infectious evaluation [26]. Additionally, a reduction of L-dopa or dopamine agonist therapy should be implemented [26]. Adjunct therapy can be provided with the addition of amantadine [26]. It should be noted that dyskinesia is not in itself a pathology but may suggest overuse of dopamine. Dyskinesia can be mitigated by screening for impulsive and addiction-like behaviors. In our experience, reduced levodopa therapy and rapid critical care intervention, result in a good prognosis. 

## 4. Hospital Related Complications

### 4.1. Aspiration Pneumonia

Dysarthria and dysphagia are common manifestations of idiopathic PD [28]. All phases of swallowing may be affected, with the oral and pharyngeal phases mostly likely to contribute to aspiration pneumonia [28]. Motor dysfunction impairs the muscles of mastication, subsequently reducing mandibular excursion and resulting in reduced mechanical agitation of food [28]. The term “tongue pumping,” which is characterized as a repetitive backward and forward rocking motion of the tongue, is pathognomonic of oral involvement in PD [28]. Irregular mechanical motion inhibits and may prevent food from being effectively swallowed, due to food not being pushed to the posterior oropharynx [28]. L-dopa causes dose-dependent xerostomia, which may be missed in the presence of sialorrhea due to difficulty controlling secretions [28]. Other mechanical aspects, such as an anteriorly flexed posture of the neck, decreased spontaneous swallowing, and delayed pharyngeal swallowing, contribute to increased aspiration risk [28]. Swallow studies have demonstrated reduced laryngeal elevation in combination with reduced pharyngeal motion resulting in cricopharyngeal dysfunction, which can reduce the appropriate passage of food boluses into the esophagus [28]. The incidence of silent aspiration is increased by an often-present weak cough [29]. A review of swallow video fluoroscopy in PD patients demonstrated as many as 18.75% experienced silent laryngeal penetration or silent aspiration [29].

#### Risk Reduction and Management

Early bedside evaluation of swallow status is important for PD patients admitted to the hospital to ensure the patient can properly hydrate, reduce pulmonary complications, and maintain proper nutrition [28]. In the event of severe dysphagia, early placement of enteral tube for medication administration should be considered [28].

L-dopa and other dopaminergic therapies improve limb motor function, though they have a less robust impact on swallowing [28]. However, it has been our experience that dopaminergic therapies do improve swallowing capabilities and should be administered to reduce aspiration pneumonia. Given the presence of dopamine related xerostomia, the patient should be encouraged to frequently consume sips of water or utilize saliva stimulating therapies (lozenges, synthetic saliva, etc.) [28].

Comorbid dementia is often present and exacerbated by hospitalization; therefore, feeding assistance should be strongly considered with appropriate upright positioning during meals [4,28]. Engaging a nutrition specialist early in the admission process to adjust the diet and discuss the importance of smaller bites, slower rates, and frequent sips of water will assist in the mitigation of aspiration [28]. Speech therapy can provide compensatory swallowing techniques, including tucking the chin to the chest or tilting the head 45 degrees to delay triggering the pharyngeal swallow, tongue base retraction, and increase airway protection [28]. Percutaneous endoscopic gastrostomy (PEG) has shown quality of life improvement via nutritional support in some PD patients [28].

Despite implementation of these mitigating measures, PD patients continue to be at an increased risk of silent aspiration, and a low threshold should be maintained to evaluate for aspiration pneumonia when there are signs of clinical decompensation, worsening motor symptoms, respiratory distress, or a fever of unknown origin [28]. It is our practice to perform annual swallow evaluation on PD patients. If a recent report is not available in the electronic medical record, we recommend a speech evaluation prior to diet initiation. Early risk identification can mitigate aspiration, which is a major cause of mortality in PD [30].

### 4.2. Urinary Tract Infections

Urinary tract infections (UTI) are a well-known cause of acute deterioration and increased morbidity in patients with Parkinson’s disease [29,30]. UTI is also a well-known cause of hospitalization for patients with PD, with an increased risk during hospitalization [31]. Unlike in the general population, PD-related UTI is prevalent equally in older men and women with known Parkinson’s. McCormack and colleagues also demonstrated that patients with PD undergoing elective procedures (e.g., total hip replacement) are at increased risk for developing an UTI while hospitalized [32]. The development of UTI is attributed to reduced motor and non-motor function (autonomic nervous system) [31]. The development of UTI further worsens these functions. The inherent disease process of PD results in degradation of the frontal-basal ganglia D1 dopaminergic circuit, inherently impairing the micturition reflex and resulting in bladder dysfunction [33].

#### Risk Reduction and Management

While hospitalized, medical personnel should exercise increased surveillance over patients with PD. Ensuring appropriate hygiene, either through direction or direct care, should be an integral part of daily rounds, especially in patients admitted with altered mental status or reduced mobility due to exacerbation of motor symptoms. Additionally, medical staff should consider scheduled bladder emptying in the case autonomic dysfunction prevents the patient from acknowledging their need to micturate. The physician should take care to ensure an adequate medication review, taking note of medications that increase urinary retention (i.e., antipsychotics/anticholinergics) or urinary incontinence (i.e., furosemide) [34]. A detailed understanding of prior UTIs or known urinary incontinence should increase the physician’s consideration for prophylactic antibiotics or clean catheterization, especially in patients with reduced mobility or altered mental status [29].

### 4.3. Falls

Falls are the most common hospital related incidents, which PD patients are at an increased risk of experiencing, especially during illness or a missed dosage. [2]. PD-specific risk factors include neurogenic/iatrogenic orthostatic hypotension, cognitive dysfunction, PD severity, prior falls, and fear of falling [2]. Impaired mobility (81%) and cognition (44%) have been shown to be present in hospitalized PD patients [2].

In a review of the general population, hospital related falls resulted in minor injuries in 40% of patients and severe injuries in 11% [2]. By comparison, 67.7% of PD patients sustained injuries related to their falls, though this study was not assessed in a hospital setting [28]. Farombi’s team suggested the Unified Parkinson’s Disease Rating Scale (UPDRS) may be an indicator for the risk of PD-related falls [35]. A statistically significant difference (*p* ≤ 0.001) was noted in the total UPDRS score when comparing PD patients who fell (UPDRS = 41.5) versus those who did not (UPDRS = 26.3) [35]. A validated fall risk assessment, such as the Tinetti balance test (71% specific and 79% sensitive), can be used as a predictor [35].

Orthostatic hypotension was reported in 52.9% of patients that fell [35]. Neurogenic orthostatic hypotension (nOH) is a well-documented manifestation of dysautonomia in PD patients, with up to 20% being symptomatic [36]. nOH increases with severity of PD, duration of PD, age, and L-dopa therapy [36]. PD patients are at a five-fold increased risk of fall related fractures, when compared to age- and sex-matched controls [37]. Risk factors include deconditioning, PD-related motor and cognitive issues, low bone mass index and mineral density, medications (i.e., sedatives), comorbidities (i.e., prior stroke), age, history of falls, and female sex [37]. In one study reviewing the outcomes of PD patients admitted to the ICU, it was identified that 12% were admitted for trauma secondary to falls, with a subset of these patients experiencing subdural hemorrhage [8].

#### Risk Reduction and Management

In addition to typical institutional fall precaution measures, early identification of motor and non-motor manifestations of PD is critical in reducing the fall risk of the hospitalized PD patient. A multi-disciplinary approach to recognizing and mitigating potential triggers such as nocturia, orthostatic hypotension, appropriate PD medication administration, and the identification of off-periods and dyskinesias should also be implemented [38]. While it is likely patients will ambulate with assistance, having accessible physical support (walkers, walking sticks, wheelchairs) and minimizing trip hazards in the room may reduce fall risk [2,38].

Cognitive impairment evaluation, bed alarms, or other preventive measures to intervene on unassisted ambulation should be implemented [2,4]. Early involvement of neurology, physical therapy, and occupational therapy to identify cognitive deficits, gait dysfunction, weakness, or difficulty with fine motor tasks may help guide the care plan to include toileting strategies or pharmacotherapy alterations that predispose PD patients to falling [2,38]. Early consultation regarding speech therapy and nutrition should be ordered to ensure optimal ability to take oral intake safely and reducing the risk of inadequate hydration and caloric intake [28]. In the event the PD patient is not tolerating enteral feeds, administration of supportive IV saline or initiation of pharmacologic support such as fludrocortisone, midodrine, or pyridostigmine can be employed to minimize the risk of symptomatic orthostatic hypotension [39]. It is our practice to perform annual fall-risk evaluation and refer for formal physical therapy gait analysis, in order to identify and mitigate falls and associated risk factors.

### 4.4. Delirium

The DSM-5 defines delirium as an acute disturbance in attention and awareness that fluctuates and is accompanied by an additional disturbance in cognition [40]. Early recognition that this is a neurologic emergency may reduce morbidity and mortality. Delirium is typically classified as hyperactive or hypoactive, depending on the clinical presentation. Prognostically, hypoactive delirium is suggestive of poor outcomes [40]. Independent risk factors include age, metabolic derangement, sleep disturbance, pain, recent surgery, or cognitive impairment [40]. No study has evaluated PD-specific delirium or whether PD patients are predisposed to delirium. However, comorbid cognitive impairment predisposes PD patients to delirium and may pose a diagnostic conundrum if patients are in a hypokinetic “off state” [40]. While fluctuating hallucinations, somnolence, and inattention can be seen in PD, acute confusion or disorientation should raise clinical suspicion for the presence of delirium [40]. Utilization of validated delirium evaluation tools such as Confusion Assessment Method (CAM), Delirium Rating Scale (DRS), or Single Question in Delirium (SQUID) with patients that have severe comorbid dementia should be employed early and often [34]. Inpatient (22–48%) and post-operative (11–60%) incidences of delirium was noted in a 2018 review [40]. Of interest, post-DBS (11–27%) rates were lower, though this is likely due to the elective nature of admission in a relatively healthier population of PD patients [39]. While evaluation of PD-specific delirium has not been conducted, studies have shown that PD is an independent risk factor for impaired clinical outcome following delirium [40]. L-dopa and PD-specific medications are known to worsen delirium [40].

#### Risk Reduction and Management

Approach to delirium management is dependent on the form (i.e., hypoactive vs. hyperactive). In hyperactive delirium, dopaminergic therapy should be reduced, though the approach is not as definitive in hypoactive delirium [40]. Rapid withdrawal of dopaminergic therapy may result in PHS. Prior to the administration of neuroleptic medications, an appropriate investigation of infection, pain, and environmental factors (i.e., sleep deprivation) should be explored [40]. Removal of medications known to contribute to delirium, such as anticholinergics, antibiotics, glucocorticoids, antidepressants, and analgesics, should be considered [40]. Initiation of melatonin for sleep-wake cycle regulation or clonazepam for nighttime confusion should also be considered [40]. If neuroleptic therapy is required, quetiapine, clozapine, and pimavanserin (serotonin 2A receptor antagonists) should be considered first-line, as these are the least likely of the typical and atypical antipsychotics to worsen PD symptoms [41]. These therapies are often limited by their lack of parenteral formulations. Lombardo and his team, in a 2020 publication, suggest ICU transfer and initiation of dexmedetomidine therapy [41]. Dexmedetomidine offers certain advantages, as it does not cause respiratory depression or worsen PD symptoms [40]. Dexmedetomidine is often utilized during DBS surgery with adverse effects [41]. Additionally, non-pharmacologic therapies such as reorientation, vision and hearing aids, nutritional support, early ambulation, and minimization of sleep cycle disturbance should be implemented, as it is estimated that 30–40% of delirium is preventable [40]. For our PD population, we recommend a thorough infectious evaluation, as it is the most common etiology of delirium in elderly PD patients.

## 5. Medication Administration Pitfalls

A general neurologist and movement disorder specialist commonly manage PD patients. In the outpatient setting, PD-specific therapy is optimized to mitigate motor manifestations, dysautonomia, cognitive dysfunction, and falls [7]. Hospital visits for PD patients often result in management by a non-specialist, with subsequent interruption of their usual medication schedule [7]. While there are many approaches to PD treatment, a typical introductory dose is levodopa-carbidopa therapy three times a day at scheduled times to maximize the therapeutic response. Crowded emergency rooms may inhibit a provider’s ability to evaluate and schedule dopaminergic therapy, resulting in missed dosages. Missing a dosage, resulting in an off period with worsened PD symptoms, may impact disposition and delay the identification of admitting etiology. Another common pitfall includes missing clinical features of Parkinson’s disease such as gait instability, increased rigidity and fall risk in undiagnosed patients resulting in failure to implement PD-specific mitigating interventions [42].

Accurate medication reconciliation and administration in PD patients will influence their hospitalization. For example, a survey of National Parkinson’s Foundation Centers concluded 71% of centers were unsure if certain antiemetic therapy was contraindicated and 80% were uncertain on antipsychotic selection to minimize PD exacerbation [7]. A study reviewing 1736 patients admitted with PD demonstrated the most commonly inappropriately administered medications were droperidol (16%), aripiprazole (15%), and promethazine [43]. Cox’s team noted that PD patients with inappropriately administered medications had a longer hospital stay [43]. While this study focused primarily on antipsychotic and antiemetic use, it also highlights challenges in medication management in PD patients. Within the same survey, 94% of centers were not confident PD patients received their PD-specific therapy on time [7]. In a review of all medications administered to 89 hospitalized PD patients (*n* = 3873), 17.4% of their medications were incorrectly administered and occurred in 80 of the 89 (89.9%) patients [44]. Of note, 7.7% of total prescribed medications were given 30 min early, and 7.9% were given 30 min late, which can significantly impact PD “on” and “off” times [44]. Hou’s team also observed that 19 of the 89 (21.3%) received pharmacotherapy that was contraindicated due to their anti-Parkinson’s therapy [44]. A review of neurologist involvement in care and medication administration found that 29 of the 89 patients had a neurologist involved, increasing the likelihood they would receive medications on time (median = 90.5% vs. 83.7%, *p* = 0.02, mean = 85.5% vs. 76.5%) [44].

Factors that did not significantly impact medication administration included day of admission, ethnicity, comorbidities, or medication schedule [44]. A separate study of 59 patients reviewing medication management in PD patients undergoing surgery demonstrated similar challenges, with 22% receiving contraindicated therapy while 12% had their PD medications omitted [45]. Hou’s team found that medication discrepancy was most likely to occur in the first 2 days of admission [44]. A separate New Zealand study demonstrated 71.9% of patients over the age of 75 taking ≥5 medications had at least one unintended discrepancy [46].

Difficulty in ascertaining correct medication reconciliation can often be confounded by comorbid cognitive deficits and the absence of a knowledgeable caregiver at the bedside [47]. Aside from medical complications related to missed PD medication administration, patients also had an increased length of stay (mean = 8.2, median 5 days) compared to patients who did not (mean = 3.6 days, median 2 days), increasing the financial burden of PD due to a preventable cause [48].

### 5.1. Risk Reduction and Management

To mitigate PD medication complications, it is imperative that extra attention is given during medication reconciliation, administration, and that thorough review of contraindications is conducted [43,44,48,49]. While electronic medical records are an excellent resource, verifying no changes since last is critical in the prevention of over or under dosing PD-related therapy [43,49]. When the patient is unable to provide their medical history, extra effort should be taken to contact a family member or physician familiar with the patient’s case to verify the dosage and administration time of PD-specific medications [37,43]. Additionally, careful attention should be taken to ensure times for administration are provided, as opposed to typical b.i.d. or t.i.d. shorthand, as this could result in inappropriate administration [43,48].

In circumstances where the patient or caregiver cannot be identified, early neurology consultation for guidance of PD therapy may be beneficial in reduction of negative outcomes [38]. Early, multi-disciplinary discussion with pharmacy and nursing staff regarding contraindicated medications (i.e., those that worsen Parkinsonism) to identify the best therapy for clinical management can reduce the risk of inappropriate administration [43,44]. Annotations in the electronic medical record should focus on time of administration, to align medication administration with home schedule [49]. Discussions with the neurologist during periods of uncertainty in the selection of antiemetic, antipsychotic, or other pharmacotherapies also provide benefit if addressed early [49]. The United Kingdom has adapted a national program to educate hospital and nursing staff about on-time administration of medications for PD, with positive outcomes demonstrating the need for raised awareness [38]. It is our practice to carefully evaluate the medication history with attention to the times of administration on every visit, in order to avoid treatment and therapy errors.

Perioperative complications of complex therapies DBS of the internal globus pallidus (GPi) and subthalamic nucleus (STN), first developed in 1986, has become a common treatment option for PD patients who are refractory to pharmacotherapy [50]. DBS has effectively demonstrated reduction of motor symptoms with many positive features, including subsequent reduction of medication requirements, minimal tissue damage, adjustable settings, and being typically reversible [50,51]. Deuschl and his team identified 25% improvement on the Parkinson’s Disease Questionnaire scale (PDQ-39) and 22% improvement on the short form survey (SF-36), in addition to UPDRS and motor symptom improvement [51]. While DBS is typically an elective surgery, implantation poses hospital-related risk for patients admitted with PD, which can result in escalation of care.

Aside from the previously mentioned complications (medication management, aspiration pneumonia, falls, delirium/cognitive impairment), special attention should be paid to pre-operative planning and perioperative management to reduce the risk of adverse effects of general anesthesia, PD exacerbation when controlling postoperative nausea and pain, frequent bladder scans for urinary retention, and dermatologic and musculoskeletal evaluation [52].

DBS surgery-specific complications include intracerebral hemorrhage (0.2–5%), postoperative infection (1.5–15.2%), and hardware complications (1.3–17.3%) [50]. Post-operative depressive and mania-like symptoms have been reported, occasionally manifesting as attempted suicide (0.9–2.0%) and completed suicide (0.45–1.0%) [50]. Factors associated with suicide include post-operative depression/apathy, being single, and having a history of impulse-control disorder [50]. Mood disturbance may be associated with STN and GPi due to stimulation of nonmotor associative or limbic circuits; however, no statistically significant difference was shown in location selection for mood manifestations [53].

DUODOPA therapy serves an alternative for patients experiencing early wearing-off, and are not candidates for DBS. To our knowledge, no studies have demonstrated superiority of DUODOPA compared to DBS. However, this therapy requires intrajejunal delivery of levodopa-carbidopa intestinal gel, increasing the risks associated with PEG-J tube placement such as intestinal obstruction, bleeding, and infection [54].

Radiofrequency and radiosurgery pallidotomies, while options, are not typical therapies currently utilized in the treatment of PD. Radiotherapy has demonstrated efficacy but requires further evaluation for tremor management. Similar to DBS, patients who undergo these procedures have an increased risk of intracerebral hemorrhage, stroke, seizures, and coma [55].

### 5.2. Risk Reduction and Management

To minimize post-operative and peri-operative complications, multiple pre-surgical and perioperative steps must be taken. Assurance that MAO-B inhibitors are stopped 1–2 weeks prior to surgery and effortful avoidance of halothane in patients on L-dopa (cardiac sensitivity to catecholamines) and propofol (dyskinesias) should be employed [49]. When managing post-operative pain, fentanyl (rigidity) should be avoided, with minimal utilization of opioid therapy when possible [49]. Postoperative nausea may pose a challenging manifestation, but metoclopramide, promethazine and prochlorperazine’s dopaminergic blocking effect can worsen PD, increasing the risk of hospital-related complications [49]. Instead antiemetics such as trimethobenzamide, ondansetron, or domperidone should be considered [49].

As PD patients may require intubation with the use of sedation, planning for sialorrhea, dysphagia, and dysmotility to reduce aspiration pneumonia should be aggressively managed with pre-surgical glycopyrrolate, ipratropium spray, or botulinum toxin type B for sialorrhea to reduce aspiration pneumonia [49]. As GI-related complications can result in ICU admission, an aggressive bowl regimen should be implemented [49]. If hyperactive delirium, mood dysfunction, or exacerbation of cognitive dysfunction is identified, atypical and typical antipsychotics, except for quetiapine, or clozapine, should be avoided due to their impact on PD symptoms [49]. Nursing staff should be directed to check for urinary retention, utilizing bladder scans as indicated, in addition to performing regular dermatologic and musculoskeletal evaluations for pressure ulcers or contractures [49]. Early physical therapy as well as hand and foot bracing may reduce the risk of contractures and ulcerations [49]. Given the likelihood for reduced mobility, deep venous thrombosis prophylaxis should be initiated to reduce and/or prevent pulmonary embolism [49]. In our practice, verifying the post-operative functionality of the DBS and prompting the initiation of L-dopa therapy has resulted in improved outcomes. We recommend a L-dopa bridge if DBS is turned off for clinical reasons.

## 6. Discussion

PD presents unique challenges during hospitalization. We provided our clinical experience and helpful suggestions with the aim of reducing mortality and morbidity through early recognition of complications, and preventative clinical interventions with a known PD patient. As the prevalence of PD continues to increase, the need for awareness of specific therapies and mitigation is imperative to reduce morbidity, mortality, and the financial burden of PD patients in the hospital. As outlined above, several aspects of PD care can be managed through early implementation of a multidisciplinary approach and careful attention to pharmacologic and non-pharmacologic management. Continued education and a multidisciplinary team can ensure a patient-centered approach to reduce the adverse outcomes of hospitalized PD patients. There are five elements when a patient presents with acute Parkinsons like symptoms that the clinician should consider, as summarized in Table 1. It is our hope that this comprehensive review be used by GME trainees and general neurologists in their care and assessment of PD patients presenting to the ER or admitted to the hospital to assist in the astute identification of toxins, medication mismanagement, and mitigation of preventable causes that contribute to morbidity and mortality in PD patients.

## Figures and Tables

**Table 1:** Pearls for the Acute evaluation of Parkinsons Disease and Parkinsons like symptoms.

If an individual presents with acute Parkinsonism, evaluate for toxic exposure.
2. An acute decline in function for PD patients should prompt a detailed medication review.
3. Avoid acute cessation of dopaminergic therapy without a planned bridge therapy.
4. Do not abruptly stop DBS therapy without a levodopa bridge therapy.
5. Always screen for dysphagia, falls, and medication administration times on every visit.
